# Case Report: Washed microbiota transplantation for the treatment of malnutrition with multidrug-resistant *Klebsiella pneumoniae* and *Candida tropicalis* coinfection in a child

**DOI:** 10.3389/fped.2026.1809311

**Published:** 2026-04-16

**Authors:** Wenkai Yang, Qian Ren, Bowen Li

**Affiliations:** 1 Department of Pediatrics, The First Hospital of Lanzhou University, Lanzhou, Gansu, China; 2 Department of Gastroenterology, The First Hospital of Lanzhou University, Lanzhou, Gansu, China

**Keywords:** *Candida tropicalis*, case report, malnutrition, multidrug-resistant *Klebsiella pneumoniae*, washed microbiota transplantation

## Abstract

**Background:**

Multidrug-resistant (MDR) *Klebsiella pneumoniae* and fungal coinfection in children with severe malnutrition are difficult to control with antibiotics alone. This report describes an 8-year-old boy whose pulmonary infection remained uncontrolled and whose nutritional status progressively deteriorated. Washed microbiota transplantation (WMT) was introduced as part of a multimodal salvage treatment strategy, after which the patient showed gradual improvement during continued antimicrobial therapy, respiratory support, and nutritional rehabilitation.

**Case presentation:**

We report the case of an 8-year-old boy with chronic malnutrition and recurrent severe pneumonia associated with an underlying central nervous system disorder. He developed recurrent respiratory failure and a persistent pulmonary infection caused by ESBL-producing MDR *K. pneumoniae* and *Candida tropicalis*. Despite broad-spectrum antimicrobial therapy, respiratory support, bronchoscopy/bronchoalveolar lavage, and enteral nutrition through a nasojejunal tube, infection control remained poor and nutritional status continued to deteriorate, complicated by sepsis and antibiotic-associated diarrhea. In this context, WMT was introduced through a nasojejunal tube as part of a multimodal salvage treatment strategy and was administered in two treatment courses. Thereafter, during continued antimicrobial treatment, respiratory support, and nutritional rehabilitation, the patient showed progressive clinical improvement, with subsequent negative sputum culture results, gradual radiographic resolution of pulmonary inflammation, weight gain from 14.0 to 22.5 kg, and marked functional recovery.

**Conclusion:**

This case suggests that, in severely malnourished children with refractory multidrug-resistant pulmonary bacterial and fungal infections, WMT may have potential adjunctive value as part of comprehensive management. However, because multiple interventions were implemented concurrently and no pre- and post-WMT microbiome sequencing was performed, the observed clinical improvement could not be attributed exclusively to WMT. Therefore, this case should be interpreted only as an exploratory clinical observation rather than confirmatory evidence, and future prospective studies under strict ethical oversight need to be conducted.

## Introduction

Multidrug-resistant (MDR) *Klebsiella pneumoniae* is an important cause of severe healthcare-associated infections in children and is associated with limited antimicrobial options and poor outcomes, particularly in patients with underlying disease, prolonged hospitalization, or malnutrition (1, 2). In recent years, increasing attention has been paid to the role of the gut microbiota in host immune regulation and systemic inflammatory homeostasis. Emerging evidence supports bidirectional interactions between the intestinal microbiota and pulmonary immunity, commonly referred to as the gut–lung axis (3–6), and therefore, microbiota-based therapies have been explored as potential adjunctive approaches in selected infectious and extraintestinal conditions (7–10).

Washed microbiota transplantation (WMT) is an optimized microbiota transplantation protocol designed to improve product safety while preserving functional microbial communities (7). Although WMT has been increasingly investigated in gastrointestinal disorders and selected systemic conditions, evidence in pediatric patients with severe malnutrition and refractory pulmonary MDR infection remains very limited (11). In particular, its role in children with concurrent multidrug-resistant bacterial and fungal pulmonary infections has not been well characterized.

Here, we report the case of an 8-year-old boy with chronic malnutrition, recurrent severe pneumonia, and an underlying central nervous system disorder. Because he developed a refractory pulmonary infection caused by ESBL-producing multidrug-resistant *K. pneumoniae* and *Candida tropicalis*, WMT was introduced as part of a multimodal salvage treatment strategy. During continued antimicrobial therapy, respiratory support, and nutritional rehabilitation, the patient subsequently showed gradual clinical improvement. As an exploratory clinical observation, this case may provide a reference for future microbiota-based intervention studies in carefully selected, high-risk pediatric patients.

## Case description

An 8-year-old boy with a history of central nervous system disorder, long-term functional impairment, and chronic malnutrition presented with recurrent severe pneumonia. Six months before his admission to our center, he had been hospitalized locally because of fever and seizures and was diagnosed with septic shock, severe pneumonia, type II respiratory failure, multidrug-resistant (MDR) *K. pneumoniae* infection, fungal infection, and status epilepticus. He underwent prolonged intensive care, including respiratory support and tracheostomy; however, the pulmonary infection remained difficult to control, and his nutritional status progressively worsened.

To evaluate possible underlying predispositions, metabolic screening performed at the Gansu Provincial Maternity and Child-Care Hospital on 12 November 2023, showed no significant abnormalities in the tested amino acids, acylcarnitines, or organic acids, and no evidence of an inborn error of metabolism was identified. In addition, whole-exome sequencing combined with mitochondrial gene testing was performed at a tertiary hospital in Beijing on 24 January 2024, and no abnormal findings were detected. Because these evaluations were completed at outside institutions, the original reports were not retained in our institutional medical record system.

**Figure 1:**
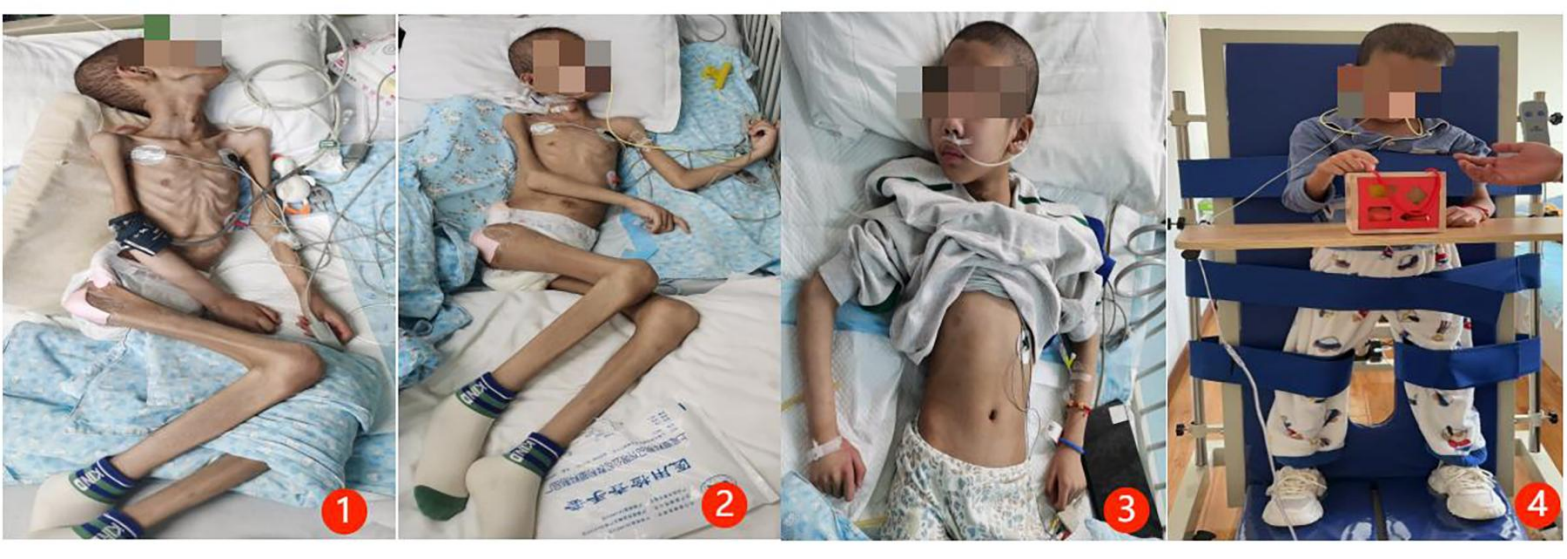
Serial photographs showing changes in nutritional status during different stages of treatment. **(1)** July 5, 2024: body weight, 14.0 kg. **(2)** July 15, 2024: body weight, 18.8 kg. **(3)** September 10, 2024: body weight, 20.0 kg. **(4)** October 12, 2024: body weight, 22.5 kg.

**Figure 2:**
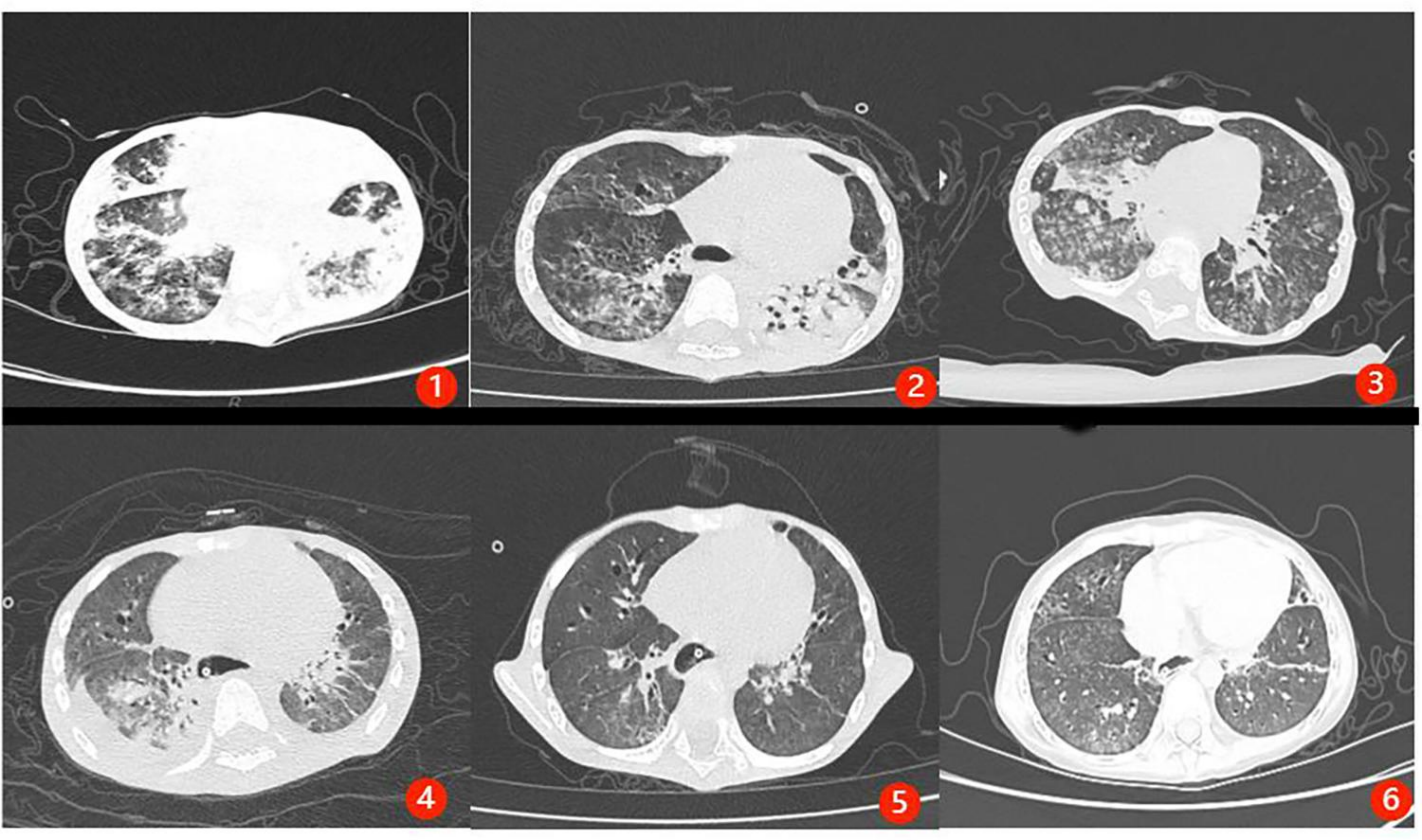
Serial chest CT changes during treatment and follow-up. **(1)** May 26, 2024: bilateral pulmonary inflammation with partial lower-lobe consolidation. **(2)** June 4, 2024: reduction in bilateral lower-lobe inflammation. **(3)** July 5, 2024: bilateral pulmonary inflammation with partial consolidation. **(4)** July 17, 2024: five days after the completion of the first WMT course: reduction in inflammatory changes in the left upper lobe and right lower lobe. **(5)** July 25, 2024: thirteen days after the completion of the first WMT course: further absorption of pulmonary inflammation. **(6)** October 28, 2024: 70 days after the completion of the two WMT courses: complete resolution of pulmonary inflammation.

After transfer and stabilization, the patient entered rehabilitation but continued to experience recurrent pulmonary infections because of severe malnutrition. Serial sputum and bronchoalveolar lavage fluid (BALF) cultures repeatedly identified ESBL-producing MDR *K. pneumoniae* and *C. tropicalis*. A chest CT revealed bilateral inflammatory infiltrates with partial consolidation. Given the repeated isolation of the same pathogens and the progressive respiratory deterioration, persistent pulmonary infection was considered the major driver of the clinical course. Serial photographs showing the patient's nutritional status at different stages during treatment and follow-up are presented in Figure 1, and serial changes in chest CT imaging during treatment and follow-up are shown in Figure 2.

### Clinical timeline

The patient's treatment course is summarized in Table 1. Briefly, between 8 May and 9 July 2024, he experienced recurrent pulmonary infection accompanied by respiratory failure, toxic shock, and severe malnutrition and was transferred to the pediatric intensive care unit. During this period, the patient required mechanical ventilation and nutritional support. Microbiological testing identified ESBL-producing *K. pneumoniae* and *C. tropicalis*. Antimicrobial treatment included cefoperazone/sulbactam plus fluconazole, followed by meropenem, caspofungin, and co-trimoxazole. Despite these interventions, the infection remained poorly controlled, with delayed pulmonary recovery, a body weight of 14.0 kg (≤3 SD), an abdominal fat thickness of 0.4 cm, and both upper- and lower-limb muscle strength graded as 1.

**Table 1:** Detailed clinical timeline of microbiological findings, concomitant interventions, WMT administration, and outcomes.

Stage and dates	Key microbiology, interventions, and outcomes
Before WMT	
8 May 2024 to 9 July 2024	Recurrent pulmonary infection with respiratory failure, toxic shock, and severe malnutrition; transfer to the PICU; mechanical ventilation and nutritional support. Microbiological testing identified ESBL-producing *K. pneumoniae* and *C. tropicalis*. Antimicrobial therapy included cefoperazone/sulbactam plus fluconazole, followed by meropenem, caspofungin, and co-trimoxazole. Despite these interventions, pulmonary recovery was delayed. Body weight was 14.0 kg, abdominal fat thickness was 0.4 cm, and both upper- and lower-limb muscle strength were grade 1.
First WMT phase	
10 July 2024 to 7 August 2024	Increased respiratory demand, moist rales, and fluctuating body temperature ranging from 37.0 °C to 39.0 °C; three WMT administrations were performed. Sputum and BALF cultures remained positive for ESBL-producing *K. pneumoniae*, and the sputum fungal culture remained positive for *C. tropicalis*. Ceftazidime plus caspofungin was administered concurrently. A follow-up chest CT revealed reduced bilateral pulmonary inflammation and absorption of pleural effusion. Body weight increased to 18.8 kg, abdominal fat thickness increased to 0.5 cm, upper-limb muscle strength improved to grade 2, and lower-limb muscle strength remained grade 1.
Second WMT phase	
7 August 2024 to 11 August 2024	Body temperature remained normal, sputum production decreased, and moist rales further reduced; three additional WMT administrations were completed. Cefoperazone/sulbactam plus caspofungin were administered concurrently. Body weight increased to 20.0 kg, abdominal fat thickness increased to 0.6 cm, and both upper- and lower-limb muscle strength improved to grade 2.
Follow-up after WMT	
12 August 2024 to 28 October 2024	The patient was afebrile, except for an occasional dry cough; continued nutritional support and rehabilitation; and no further antibiotic therapy was required. The sputum bacterial culture results were negative. Pulmonary infection was considered controlled, and chest CT results returned to normal by 2024-10-28. Body weight increased to 22.5 kg, abdominal fat thickness increased to 0.7 cm, upper-limb muscle strength recovered to grade 5, and lower-limb muscle strength improved to grade 4.

Between 10 July and 7 August 2024, the patient underwent the first WMT treatment course, which consisted of three consecutive daily administrations. At the beginning of this stage, he had increased respiratory demand, moist rales, inability to inhale effectively, and fluctuating body temperature between 37.0 °C and 39.0 °C. Sputum and BALF cultures remained positive for ESBL-producing *K. pneumoniae*, and the sputum fungal culture remained positive for *C. tropicalis*. Concomitant treatment with ceftazidime plus caspofungin was continued. By 15 July, the patient’s body temperature had normalized, the cough had reduced, and a follow-up chest CT demonstrated decreased bilateral pulmonary inflammation and absorption of pleural effusion. During this phase, the body weight increased to 18.8 kg, abdominal fat thickness increased to 0.5 cm, upper-limb muscle strength improved to grade 2, and lower-limb strength remained grade 1.

Between 7 August and 11 August 2024, because the patient's pulmonary infection and microbiota dysbiosis had not fully resolved after the first course of WMT, a second course of washed microbiota transplantation was administered, consisting of daily treatments for three consecutive days. During this period, the patient remained afebrile, sputum production decreased, and moist rales further reduced. Concomitant antimicrobial therapy with cefoperazone/sulbactam and caspofungin was administered. Nutritional status and motor function continued to improve, with the body weight reaching 20.0 kg, abdominal subcutaneous fat thickness measuring 0.6 cm, and both upper- and lower-limb muscle strength recovering to grade 2.

During the follow-up period from 12 August to 28 October 2024, the patient remained afebrile, except for an occasional dry cough, and continued to receive nutritional support and rehabilitation exercises. No additional antibiotics were required. The sputum bacterial culture results were negative, and by 28 October 2024, the pulmonary infection was considered controlled, with a chest CT showing complete resolution of bilateral inflammatory changes. At that time, the patient’s body weight had increased to 22.5 kg (≤1 SD), abdominal fat thickness to 0.7 cm, upper-limb muscle strength had recovered to grade 5, and lower-limb muscle strength had improved to grade 4.

### Therapeutic intervention

Given the poor infection control despite repeated broad-spectrum antimicrobial therapy, respiratory support, bronchoscopic airway clearance/bronchoalveolar lavage, and combined enteral and parenteral nutritional support, WMT was introduced as part of a multimodal salvage treatment strategy after a multidisciplinary discussion.

WMT was administered through a nasojejunal tube. Donor fecal samples were obtained from the China Fecal Microbiota Bank and rigorously screened according to previously published standardized criteria. WMT is an optimized fecal microbiota transplantation protocol based on a washed microbiota preparation. In this study, the microbiota product was prepared according to a standardized protocol. As previously described, washed microbiota preparation is based on an automatic microfiltration system combined with repeated centrifugation and resuspension procedures under dedicated processing conditions, aiming to reduce fecal impurities while maintaining functional microbiota components (7).

The patient underwent WMT through nasojejunal tube infusion at a dose of 1 U per administration (approximately 1.0 × 10^13^ bacterial cells), once daily for 3 consecutive days per course, completing a total of 2 courses. The first course was administered from 10 July to 12 July 2024, and the second course from 7 August to 9 August 2024. During this period, comprehensive treatment, including anti-infective therapy, respiratory support, and nutritional rehabilitation, was continued according to the patient's clinical condition. No obvious WMT-related adverse events were observed during treatment.

### Follow-up and outcomes

Clinical outcomes were assessed using serial evaluations of body temperature, respiratory symptoms, microbiological culture results, chest CT findings, nutritional status, and motor function. During the first treatment phase, the patient's body temperature gradually normalized, cough and sputum production decreased, and a follow-up chest CT showed partial absorption of bilateral pulmonary inflammation and pleural effusion. During the second treatment phase, sputum volume further decreased and moist rales reduced.

A microbiological follow-up revealed that the sputum bacterial culture results were negative during follow-up after completion of the second WMT course. By 28 October 2024, a chest CT showed complete resolution of bilateral pulmonary inflammatory changes, and no additional antibiotics were required during the follow-up period.

Nutritional and functional recovery was also observed over time. Body weight increased from 14.0 kg before WMT to 18.8 kg during the first treatment phase, 20.0 kg after the second phase, and 22.5 kg at follow-up. Abdominal fat thickness increased from 0.4 to 0.5 cm, 0.6 cm, and finally, 0.7 cm. Upper-limb muscle strength improved from grade 1 to grade 2, grade 2, and eventually grade 5, while lower-limb muscle strength improved from grade 1 to grade 1, grade 2, and then grade 4. The patient regained assisted ambulation, and his joint mobility and standing ability improved substantially. No obvious WMT-related adverse events were observed during the follow-up period.

In this patient, WMT was introduced as part of the overall treatment strategy; however, because multiple therapeutic interventions were administered concurrently, the observed clinical improvement could not be attributed entirely to WMT.

## Discussion

Multidrug-resistant (MDR) pulmonary infection in children, especially in the presence of severe malnutrition, underlying neurological dysfunction, prolonged immobility, and repeated exposure to broad-spectrum antibiotics, are extremely difficult to manage (1, 2). In the present case, the patient had persistent ESBL-producing *K. pneumoniae* and *C. tropicalis* coinfection, despite undergoing repeated antimicrobial treatments, respiratory support, bronchoscopic intervention, and nutritional therapy. This clinical course highlights the complexity of managing refractory infections in severely debilitated children and the limited effectiveness of conventional treatment alone in some high-risk settings.

Malnutrition likely played an important role in the persistence of the infection in this patient. Increasing evidence suggests that prolonged nutritional deprivation may alter microbial ecology, impair host defense, and contribute to persistent colonization or overgrowth of opportunistic pathogens, including *Klebsiella* species (12, 13). In addition, repeated and prolonged exposure to broad-spectrum antimicrobials may further disrupt colonization resistance and select for persistent multidrug-resistant organisms (3, 4). In this context, the combination of severe malnutrition, recurrent pulmonary infection, prolonged critical illness, and antibiotic exposure may have created a self-reinforcing cycle of dysbiosis, immune dysfunction, and impaired clinical recovery.

The rationale for considering microbiota-based therapy in this setting is biologically plausible but remains incompletely understood. The gut microbiota plays an important role in immune maturation, inflammatory regulation, and metabolic homeostasis (5). Experimental and clinical studies have suggested bidirectional interactions along the gut–lung axis, whereby intestinal microbiota and their metabolites may influence pulmonary immune responses (2, 5, 6, 8). In this framework, fecal microbiota transplantation (FMT) and, more recently, WMT, have been explored for selected intestinal and extraintestinal disorders (7, 14). Some preliminary reports have suggested that microbiota-based interventions may be associated with improved recovery from severe infection, antibiotic-associated dysbiosis, and selected MDR-related conditions (8–10). However, evidence in pediatric patients remains limited, particularly in critically ill children with extraintestinal infections (11).

In the present case, WMT was introduced only after conventional treatment had failed to achieve satisfactory infection control and nutritional status continued to worsen. Importantly, the clinical improvement observed in this patient cannot be exclusively attributed to WMT. Multiple concomitant interventions were administered during the same period, including broad-spectrum antimicrobial therapy, respiratory support, bronchoscopy/bronchoalveolar lavage, and intensive nutritional rehabilitation. Therefore, the patient's recovery should be interpreted in the context of multimodal treatment rather than as evidence of a stand-alone therapeutic effect of WMT.

Similarly, although the gut–lung axis provides a possible conceptual framework for understanding why microbiota-based intervention might be relevant in pulmonary infection (5, 6, 15, 16), this mechanism was not directly demonstrated in our patient. A major limitation of this study is that pre- and post-WMT microbiome sequencing, metabolomic analyses, and other direct assessments of microbial function were not performed. During clinical treatment, a fecal bacterial smear examination showed marked dysbiosis before WMT, manifested by a severely reduced total bacterial count, the presence of only Gram-positive cocci, and a cocci-to-bacilli ratio of 7:3. After microbiota transplantation, the total bacterial count and cocci-to-bacilli ratio returned to a more balanced pattern, suggesting a possible improvement in intestinal microbial homeostasis. However, because a more comprehensive microbiome characterization was not performed, we were unable to confirm that microbiota reconstruction had occurred, nor could we demonstrate that the observed clinical improvement was mediated by microbiota modulation.

Another important consideration is the safety and regulatory context of FMT/WMT in children. Microbiota-based therapy in pediatric patients requires exercising caution, especially in those with severe debility, complex comorbidities, or critical illness (7, 9). Current recommendations emphasize careful donor screening, standardized product preparation, strict procedural quality control, and close monitoring for adverse events (9, 10). Potential risks include infectious transmission, procedure-related complications, and uncertain long-term effects on the developing host. Therefore, this case should not be interpreted as supporting the routine use of WMT for pediatric pulmonary infection. Rather, it represents a carefully considered salvage intervention undertaken in a life-threatening clinical context after a multidisciplinary assessment and failure of conventional therapy.

This study has several additional limitations. First, this is a single case report and therefore cannot establish efficacy. Second, because some metabolic and genetic evaluations were completed at outside institutions, the source reports were not retained in our institutional medical record system. Third, although serial clinical outcomes, microbiological results, chest CT findings, and nutritional recovery were documented, some biochemical, inflammatory, and metabolite-related data were not retrospectively available. These limitations reduce the strength of causal inference and underscore the exploratory nature of this report.

Despite these limitations, this case report may still have clinical value. It provides real-world observational evidence that WMT may be a feasible adjunctive rescue strategy in severely malnourished children with refractory MDR pulmonary infection and fungal coinfection. More importantly, it highlights the need for future prospective studies incorporating standardized clinical outcomes, longitudinal microbiome characterization, metabolomic profiling, and longer follow-up times to better define the safety, feasibility, and potential role of WMT in carefully selected pediatric patients.

## Conclusion

Pulmonary infections caused by multidrug-resistant organisms in children, particularly those with severe malnutrition, underlying disease, and complex clinical conditions, remain difficult to manage and are associated with substantial risks. When conventional antimicrobial and supportive treatments fail to achieve adequate control, WMT may be considered a potential adjunct in a multimodal salvage strategy.

In the present case, the patient showed progressive improvement in infection control, nutritional status, and functional recovery during comprehensive management, which included antimicrobial therapy, respiratory support, bronchoscopic intervention, and nutritional rehabilitation.

Importantly, the observed clinical improvement cannot be solely attributed to WMT. In addition, owing to the absence of pre- and post-WMT microbiome or metabolomic data, this study does not demonstrate microbiota reconstruction or confirm a gut-lung axis-mediated mechanism. Rather, this case provides exploratory real-world evidence that may help inform future investigations.

Prospective studies incorporating standardized clinical outcomes, longitudinal microbiome characterization, metabolomic analysis, and longer-term follow-up times are warranted to further evaluate the safety, feasibility, and potential role of WMT in pediatric patients with severe infection and malnutrition.

## Patient perspective

The patient's parents reported that his fever and diarrhea reduced soon after WMT and that his appetite and physical strength gradually increased during rehabilitation. They expressed satisfaction with the overall recovery and willingness to continue with follow-up.

## Data Availability

The original contributions presented in this study are included in the article/Supplementary Material, and further inquiries can be directed to the corresponding author.
