# Supplementary material for: Case Report: Washed microbiota transplantation for the treatment of malnutrition with multidrug-resistant Klebsiella pneumoniae and Candida tropicalis coinfection in a child
Source: Front Pediatr. 2026 Apr 16;14:1809311. doi: 10.3389/fped.2026.1809311 (PMC13128628; doi:10.3389/fped.2026.1809311)
Supplement: Supplementary file 1 [file Table1.docx]

| **Item** | **Time Period** | **Main Disease and Treatment Measures** | **Pulmonary Infection** | **Antibiotic Application** | **Pulmonary Infection Outcomes** | **Nutritional Status** | **Muscle Strength** |
| --- | --- | --- | --- | --- | --- | --- | --- |
| **Before WMT** | 2024.05.08–2024.07.09 | Fever, cough, difficulty breathing, recurrent pulmonary infections with combined respiratory failure, toxic shock, poor nutritional status, transferred to PICU. Supported with mechanical ventilation and external nutritional support; bacterial culture was positive. | ESBL+ Klebsiella pneumoniae, Candida tropicalis infections. | cefoperazone/sulbactam + Fluconazole; Meropenem + Caspofungin + Co-trimoxazole | Bilateral pneumonia; 7.9 day after respiratory support via endotracheal tube, no significant fibrosis, infection delayed. | Weight 14.0 kg (< -3 SD), abdominal fat thickness 0.4 cm. | Upper limb strength Grade 1, lower limb strength Grade 1. |
| **First Week of WMT** | 2024.07.10–2024.08.07 | Increased respiratory demand, lung moist rales and inability to inhale, body temperature fluctuating between 37.0–39.0°C; underwent 3 WMT sessions. Improvement noted on 7.15 with temperature normalization and reduced coughing. | Sputum/BALF Culture: Klebsiella pneumoniae (ESBL+)；  Sputum Fungal Culture: Candida tropicalis | Ceftazidime + Caspofungin | 7.15 follow-up chest CT showed reduced bilateral lung inflammation and absorption of pleural effusion. | Weight 16.0 kg (< -3 SD), abdominal fat thickness 0.5 cm. | Upper limb strength Grade 2, lower limb strength Grade 1. |
| **Second Week of WMT** | 2024.08.07–2024.08.11 | Normal body temperature, reduced sputum, moist rales decreased; underwent 3 additional WMT sessions. | - | cefoperazone/sulbactam + Caspofungin | - | Weight 20.0 kg (< -2 SD), abdominal fat thickness 0.6 cm. | Upper limb strength Grade 2, lower limb strength Grade 2. |
| **After WMT** | 2024.08.12–2024.10.28 | Normal body temperature, occasional dry cough, continued nutritional support and rehabilitation exercises. | Sputum Bacterial Culture: Negative | No antibiotics | 10.28 Pulmonary infection under control, chest CT normal by 10.28. | Weight 22.5 kg (< -1 SD), abdominal fat thickness 0.7 cm. | Upper limb strength Grade 5, lower limb strength Grade 4. |

Table 1 summarizes key clinical events, microbiology, interventions, and outcomes during the episode of care.
