# Supplementary material for: Case Report: Washed microbiota transplantation for the treatment of malnutrition with multidrug-resistant Klebsiella pneumoniae and Candida tropicalis coinfection in a child
Source: Front Pediatr. 2026 Apr 16;14:1809311. doi: 10.3389/fped.2026.1809311 (PMC13128628; doi:10.3389/fped.2026.1809311)
Supplement: Supplementary file 2 [file Table2.docx]

**CARE Checklist (Completed) – Case Report Submission**

Manuscript title: Case Report: Washed Microbiota Transplantation for the Treatment of Malnutrition with Multidrug-Resistant Klebsiella pneumoniae and Candida tropicalis Co-infection in a Child

Please indicate where each item is reported in the manuscript (section/paragraph).

| Item | Checklist description | Reported in manuscript (section) |
| --- | --- | --- |
| 1 | Title includes the words “case report” and the area of focus | Title |
| 2 | 2–5 keywords, including “case report” | Keywords |
| 3a–d | Abstract: introduction, case presentation, and conclusions (no references) | Abstract |
| 4 | Introduction: what is unique, relevant literature | Introduction |
| 5a–d | Patient information: de-identified info, main concerns, history, past interventions | Case description |
| 6 | Clinical findings (physical exam, clinical findings) | Case description |
| 7 | Timeline | Timeline/Table 1 |
| 8a–d | Diagnostic assessment: tests, challenges, diagnosis/differential, prognosis | Diagnostic assessment |
| 9a–c | Therapeutic intervention: type, administration, changes | Therapeutic intervention |
| 10a–d | Follow-up and outcomes: outcomes, follow-up, adherence, adverse events | Follow-up and outcomes |
| 11a–d | Discussion: strengths/limitations, literature, rationale, take-away message | Discussion |
| 12 | Patient perspective | Patient perspective |
| 13 | Informed consent | Ethics statement |
